# Supplementary material for: Dazzling damselfish: investigating motion dazzle as a defence strategy in humbug damselfish (Dascyllus aruanus)
Source: PeerJ. 2024 Sep 25;12:e18152. doi: 10.7717/peerj.18152 (PMC11438442; doi:10.7717/peerj.18152)
Supplement: Supplemental Information 1 [file peerj-12-18152-s001.docx]

**Supplemental Information**

Dazzling damselfish: Investigating motion dazzle as a defence strategy in humbug damselfish (*Dascyllus aruanus*)

Louise Tosetto, Nathan S. Hart & Laura A. Ryan

Corresponding author: Louise Tosetto – louise.tosetto@mq.edu.au

This file includes:

Figure S1

Figure S2

Figure S3

Table S1

Table S2

Table S3

Table S4


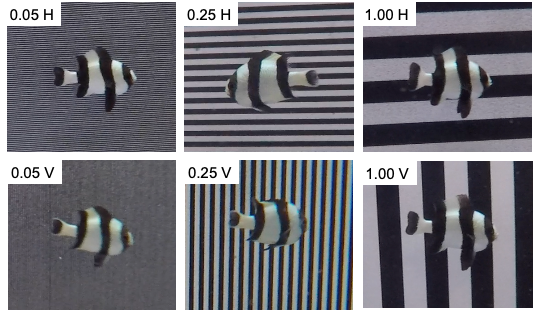


Figure S1.Screen shots from videos used in the motion cue modelling. These screen shots show example backgrounds with humbugs for the 0.05, 0.25 and 1.00 cm gratings at horizontal and vertical orientation (Source Credit: Tosetto et al. 2024)

Figure S2. Mean (+/- SE) distance that humbugs were positioned in relation to the different gratings over the five-minute trial (n = 20).

Figure S3. Mean (+/- SE) distance that humbugs moved in relation to the different gratings over the five minute trial (n = 20).

Table S1. Pairwise comparisons for the contrast between the horizontal and vertical orientations for each of the different gratings (cm) for the mean distance fish were positioned from the gratings every 15 seconds. Significant contrasts in bold.

| **Grating (cm)** | **contrast** | **estimate** | **SE** | ***z*** | ***P*** |
| --- | --- | --- | --- | --- | --- |
| 0.05 | H - V | 0.163 | 0.079 | 2.064 | 0.039 |
| 0.10 | H - V | 0.053 | 0.080 | 0.672 | 0.502 |
| 0.25 | H - V | 0.045 | 0.080 | 0.554 | 0.580 |
| **0.50** | **H - V** | **0.620** | **0.082** | **7.602** | **<0.001** |
| 1.00 | H - V | 0.078 | 0.079 | 0.988 | 0.323 |

Provides estimated marginal means (estimate) for contrasts between horizontal and vertical orientations for each grating width. Also provides the standard error (SE), test-statistic – z-score (*z*) and P values (*P*) are reported. *P* < 0.05 level of significance

Table S2. Pairwise comparisons for the contrast between the grating sizes for the different orientations for the mean distance fish were positioned from the gratings every 15 seconds. Significant contrasts in bold.

| **Orientation** | **contrast** | **estimate** | **SE** | ***z*** | ***P*** |
| --- | --- | --- | --- | --- | --- |
| H | **0.05 - 0.10** | **0.280** | **0.080** | **3.498** | **0.004** |
|  | **0.05 - 0.25** | **0.333** | **0.080** | **4.150** | **<0.001** |
|  | 0.05 - 0.50 | 0.047 | 0.081 | 0.578 | 0.978 |
|  | **0.05 - 1.00** | **0.351** | **0.080** | **4.412** | **<0.001** |
|  | 0.10 - 0.25 | 0.053 | 0.080 | 0.656 | 0.966 |
|  | **0.10 - 0.50** | **-0.233** | **0.081** | **-2.879** | **0.033** |
|  | 0.10 - 1.00 | 0.071 | 0.080 | 0.888 | 0.901 |
|  | **0.25 - 0.50** | **-0.286** | **0.081** | **-3.530** | **0.004** |
|  | 0.25 - 1.00 | 0.018 | 0.080 | 0.226 | 0.999 |
|  | **0.50 - 1.00** | **0.304** | **0.080** | **3.781** | **0.001** |
| V | 0.05 - 0.10 | 0.170 | 0.078 | 2.171 | 0.191 |
|  | 0.05 - 0.25 | 0.214 | 0.079 | 2.703 | 0.054 |
|  | **0.05 - 0.50** | **0.504** | **0.080** | **6.329** | **<0.001** |
|  | **0.05 - 1.00** | **0.266** | **0.079** | **3.373** | **0.007** |
|  | 0.10 - 0.25 | 0.044 | 0.080 | 0.551 | 0.982 |
|  | **0.10 - 0.50** | **0.333** | **0.080** | **4.167** | **<0.001** |
|  | 0.10 - 1.00 | 0.096 | 0.079 | 1.210 | 0.746 |
|  | **0.25 - 0.50** | **0.289** | **0.081** | **3.587** | **<0.001** |
|  | 0.25 - 1.00 | 0.052 | 0.080 | 0.648 | 0.967 |
|  | **0.50 - 1.00** | **-0.237** | **0.080** | **-2.953** | **0.026** |

Provides estimated marginal means (estimate) for contrasts between gratings for horizontal and vertical orientations. Also provides the standard error (SE), test-statistic – z-score (*z*) and P values (*P*) are reported. *P* < 0.05 level of significance

Table S3. Pairwise comparisons for the contrast between the horizontal and vertical orientations for each of the different gratings for the mean distance (cm) that fish moved every 15 seconds. Significant contrasts in bold.

| **Grating (cm)** | **contrast** | **estimate** | **SE** | ***z*** | ***P*** |
| --- | --- | --- | --- | --- | --- |
| **0.05** | **H - V** | **-0.667** | **0.071** | **-9.345** | **<0.001** |
| **0.10** | **H - V** | **-0.263** | **0.071** | **-3.688** | **<0.05** |
| **0.25** | **H - V** | **0.278** | **0.071** | **3.896** | **<0.05** |
| **0.50** | **H - V** | **0.203** | **0.071** | **2.846** | **0.004** |
| **1.00** | **H - V** | **-0.198** | **0.071** | **-2.77** | **0.006** |

Provides estimated marginal means (estimate) for contrasts between horizontal and vertical orientations for each grating width. Also provides the standard error (SE), test-statistic – z-score (*z*) and P values (*P*) are reported. *P* < 0.05 level of significance

Table S4. Pairwise comparisons for the contrast between the different gratings for each of the different orientations for the mean distance (cm) that fish moved every 15 seconds.

| **Orientation** | **contrast** | **estimate** | **SE** | ***z*** | ***P*** |
| --- | --- | --- | --- | --- | --- |
| **H** | **0.05 - 0.10** | **-0.234** | **0.071** | **-3.277** | **0.009** |
|  | **0.05 - 0.25** | **-0.415** | **0.071** | **-5.811** | **<0.001** |
|  | **0.05 - 0.50** | **-0.427** | **0.071** | **-5.978** | **<0.001** |
|  | **0.05 - 1.00** | **-0.346** | **0.071** | **-4.854** | **<0.001** |
|  | 0.10 - 0.25 | -0.181 | 0.071 | -2.534 | 0.083 |
|  | 0.10 - 0.50 | -0.193 | 0.071 | -2.701 | 0.054 |
|  | 0.10 - 1.00 | -0.113 | 0.071 | -1.577 | 0.512 |
|  | 0.25 - 0.50 | -0.012 | 0.071 | -0.168 | 1.000 |
|  | 0.25 - 1.00 | 0.068 | 0.071 | 0.957 | 0.874 |
|  | 0.50 - 1.00 | 0.080 | 0.071 | 1.124 | 0.794 |
| **V** | 0.05 - 0.10 | 0.170 | 0.071 | 2.380 | 0.121 |
|  | **0.05 - 0.25** | **0.530** | **0.071** | **7.431** | **<0.001** |
|  | **0.05 - 0.50** | **0.443** | **0.071** | **6.212** | **<0.001** |
|  | 0.05 - 1.00 | 0.123 | 0.071 | 1.721 | 0.421 |
|  | **0.10 - 0.25** | **0.360** | **0.071** | **5.050** | **<0.001** |
|  | **0.10 - 0.50** | **0.274** | **0.071** | **3.832** | **<0.001** |
|  | 0.10 - 1.00 | -0.047 | 0.071 | -0.659 | 0.965 |
|  | 0.25 - 0.50 | -0.087 | 0.071 | -1.218 | 0.741 |
|  | **0.25 - 1.00** | **-0.408** | **0.071** | **-5.710** | **<0.001** |
|  | **0.50 - 1.00** | **-0.321** | **0.071** | **-4.491** | **<0.001** |

Provides estimated marginal means (estimate) for contrasts between gratings for horizontal and vertical orientations. Also provides the standard error (SE), test-statistic – z-score (*z*) and P values (*P*) are reported. *P* < 0.05 level of significance
